# PepN is a non-essential, cell wall-localized protein that contributes to neutrophil elastase-mediated killing of *Streptococcus pneumoniae*

**DOI:** 10.1371/journal.pone.0211632

**Published:** 2019-02-01

**Authors:** Charmaine N. Nganje, Scott A. Haynes, Christine M. Qabar, Rachel C. Lent, Elsa N. Bou Ghanem, Mara G. Shainheit

**Affiliations:** 1 Department of Biological Sciences, Towson University, Towson, MD, United States of America; 2 Department of Microbiology and Immunology, University at Buffalo School of Medicine, Buffalo, NY, United States of America; Louisiana State University, UNITED STATES

## Abstract

*Streptococcus pneumoniae* (*Spn*) is an asymptomatic colonizer of the human nasopharynx but can also cause disease in the inner ear, meninges, lung and blood. Although various mechanisms contribute to the effective clearance of *Spn*, opsonophagocytosis by neutrophils is perhaps most critical. Upon phagocytosis, *Spn* is exposed to various degradative molecules, including a family of neutrophil serine proteases (NSPs) that are stored within intracellular granules. Despite the critical importance of NSPs in killing *Spn*, the bacterial proteins that are degraded by NSPs leading to *Spn* death are still unknown. In this report, we identify a 90kDa protein in a purified cell wall (CW) preparation, aminopeptidase N (PepN) that is degraded by the NSP neutrophil elastase (NE). Since PepN lacked a canonical signal sequence or LPxTG motif, we created a mutant expressing a FLAG tagged version of the protein and confirmed its localization to the CW compartment. We determined that not only is PepN a CW-localized protein, but also is a substrate of NE in the context of intact *Spn* cells. Furthermore, in comparison to wild-type TIGR4 *Spn*, a mutant strain lacking PepN demonstrated a significant hyper-resistance phenotype *in vitro* in the presence of purified NE as well as in opsonophagocytic assays with purified human neutrophils *ex vivo*. Taken together, this is the first study to demonstrate that PepN is a CW-localized protein and a substrate of NE that contributes to the effective killing of *Spn* by NSPs and human neutrophils.

## Introduction

*Streptococcus pneumoniae* (*Spn*) is a Gram-positive bacterium that is a frequent, asymptomatic colonizer of the human upper respiratory tract. However, if it gains access to other anatomical sites in the human host, such as the lungs, inner ear, meninges or blood, it can cause a variety of diseases including pneumonia, otitis media, meningitis and sepsis, respectively [1–4]. Due to these invasive infections, about one million children die per year under the age of five, mostly in the developing world where access to healthcare is limited [5]. Neutrophils are the most abundant white blood cell in the body and are often the first immune cell type to migrate to the site of infection [6, 7]. Neutrophils play a critical role in the effective clearance of *Spn* via the process of opsonophagocytic killing. This multi-step process involves the tagging of *Spn* cells with complement proteins and subsequent internalization and degradation through the action of various factors including reactive oxygen and nitrogen species, antimicrobial peptides and a family of enzymes contained within the azurophilic granules, neutrophil serine proteases (NSPs) [8]. Of this repertoire of anti-microbial factors, previous work demonstrated that NSPs are the most important component for effectively killing *Spn in vitro* [9] and play a vital, protective role in murine models of pneumococcal pneumonia [10]. Furthermore, in individuals with Chediak-Higashi syndrome, a rare genetic disorder that impairs the mobilization of NSP-containing granules [11], neutrophils exhibited a reduced ability to kill *Spn* [12].

To date, four enzymes have been identified as members of the NSP family: neutrophil elastase (NE), cathepsin G (CG), proteinase 3 (PR3) and neutrophil serine protease 4 (NSP4) [13, 14]. NSPs are members of the chymotrypsin family of serine proteases and contain a His-Asp-Ser catalytic triad [14, 15]. NSPs become enzymatically active when NSP-containing granules fuse with the phagocytic compartment and can also be exocytosed as a component of neutrophil extracellular traps (NETs) to combat extracellular pathogens [16, 17]. However, previous studies demonstrated that *Spn* can persist within NETs and thus this mechanism is not believed to be responsible for killing during infection [18, 19]. Rather, the dominant means of clearing *Spn* seems to be opsonsophagocytosis. Several studies revealed that NSPs can reduce bacterial pathogenicity by degrading virulence factors produced by a range of pathogens, including *Shigella flexneri*, *Salmonella enterica* serovar Typhimurium, *Yersinia enterocolitica* and *Staphylococcus aureus* [20, 21]. In addition, NSPs have been shown to directly kill *Pseudomonas aeruginosa*, *Escherichia coli* and *Klebsiella pneumoniae* [22–25]. Specifically, in *E*. *coli*, it was revealed that NE degrades OmpA, which destabilized the cell and induced cell death [22, 23]. Additionally, NE-mediated degradation of OprF, a major outer membrane protein in *P*. *aeruginosa*, was demonstrated to be necessary for effective immune defense in a mouse model of lung infection [24]. These findings emphasize the importance of NSPs in anti-microbial defenses and that they achieve this, in part, by degrading specific bacterial proteins. Although the importance of NSPs in controlling *Spn* infection is well established [9, 10], the exact surface proteins on this pathogen that are degraded by NSPs leading to *Spn* death have yet to be identified.

In this study, we aimed to identify specific CW-localized *Spn* proteins that are degraded by NE and/or CG, since these two NSPs were shown to be important for killing *Spn* both *in vitro* and *in vivo* [9, 10]. In experiments using a purified CW preparation, we identified a ~90kDa protein that was specifically and significantly degraded by purified NE. Analysis by mass spectrometry revealed this protein to be aminopeptidase N (PepN), an annotated metalloproteinase predicted to cleave a variety of peptides from the N-terminus [26]. Since PepN lacked an obvious secretion signal or CW localization motif, we epitope-tagged the C-terminus and performed sub-cellular fractionation experiments that revealed that PepN did indeed localize to the CW compartment. Importantly, PepN was shown to be a substrate of purified NE both in experiments with purified CW and in intact *Spn* cells. Furthermore, a mutant strain of *Spn* lacking PepN (Δ*pepN*) was significantly more resistant to killing by purified NE and opsonophagocytic killing by human neutrophils. Taken together, these data identify the first *Spn* cell wall protein that is degraded by the NSP, neutrophil elastase, and demonstrate that the degradation of PepN contributes to the effective killing of *Spn*.

## Materials and methods

### Bacterial strains, growth conditions and growth curves

*S*. *pneumoniae* strain TIGR4 (serotype 4) was used throughout this study. *S*. *pneumoniae* was grown in Todd-Hewitt broth (BD) supplemented with 0.5% yeast extract (Fisher) (THY) and oxyrase (5μL/mL) or trypticase soy broth (TSB) (BD) supplemented with catalase (Sigma; 30U/mL). Alternatively, bacteria were grown on tryptic soy agar (TSA) supplemented with 200U/mL of catalase. Where appropriate during growth, antibiotics at the following concentrations were included: chloramphenicol (Cm; 4μg/mL) or streptomycin (Sm; 100μg/mL). All cells were grown at 37°C in a 5% CO_2_ incubator.

For growth curve experiments, strains of interest were grown to mid-log phase (OD_600_ ~0.6) in THY supplemented with oxyrase and then back-diluted to an OD_600_ ~0.003 in fresh media. Next, 200μL of cells were added to 96 well flat bottom plates in replicates of 8 wells per strain and were incubated at 37°C for 12h. OD_600_ measurements were taken every 30 minutes using a VersaMax plate reader.

### Generation of mutant strains

**Fig 1:**
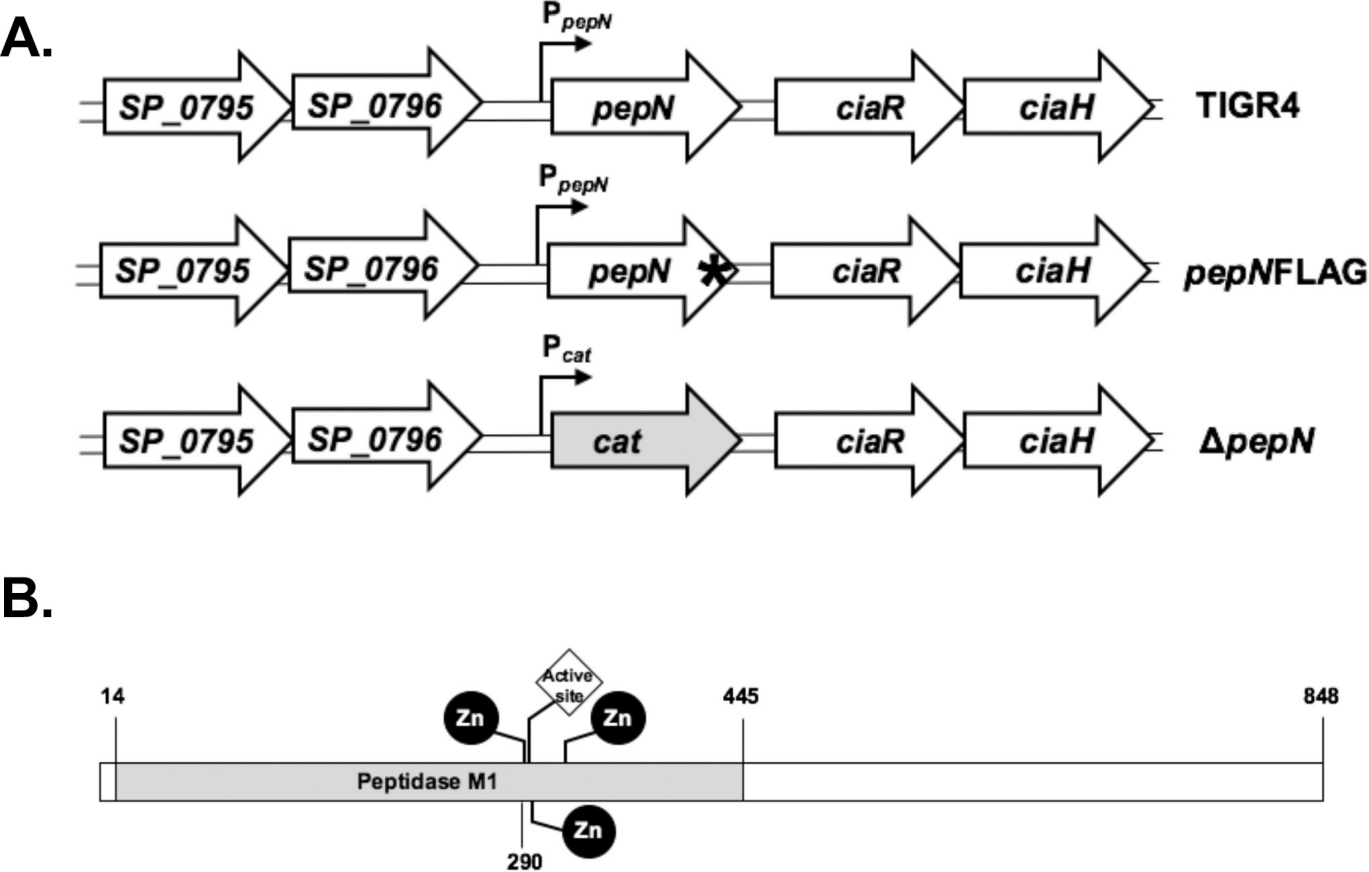
Schematic of DNA constructs used to generate *Spn* mutant strains and the domain structure of the PepN protein. (**A)** The depicted DNA constructs were used to create mutant strains of *S*. *pneumoniae* via allelic exchange. In the *ΔpepN* strain, the *pepN* ORF was replaced by a Cm resistance cassette. In the *pepN*FLAG mutant a 1X FLAG tag (denoted by *) was added to the C terminus of the PepN protein, immediately upstream of the stop codon. **(B)** The domain structure of PepN was determined using BLASTP to be comprised of a peptidase M1 domain containing an active site coordinated by three zinc binding sites (residues 292, 296 and 315).

All mutant strains of *S*. *pneumoniae* used in this study are described in **Table 1** and were generated by allelic exchange using the DNA constructs shown in **Fig 1A.** Each allelic exchange construct was generated *in vitro* using splice by overlap extension PCR [27]. To create the *ΔpepN* mutant, ~1.5kb arms of homology flanking the *pepN* gene were PCR amplified from TIGR4 genomic DNA (gDNA). The Cm^R^ cassette was amplified from pAC100 [28] and incorporated into this construct to replace the *pepN* gene, which allowed for the direct selection of transformants. To generate the *ΔpepN*Revertant strain, the wild-type *pepN* gene plus ~3kb of flanking DNA sequence was PCR amplified from TIGR4 gDNA. Subsequently, using the *ΔpepN* mutant as the recipient strain, we performed co-transformation with the revertant DNA construct and the mutant *rpsL* allele (Sm^R^) and screened for Cm^S^Sm^R^ transformants. We also used co-transformation to make the PepNFLAG tag strain, which introduced a 1X FLAG epitope directly upstream of the stop codon in the *pepN* open reading frame [29, 30]. The constructs used to generate the *ΔpepN* and *Δ*pepNRev strains are illustrated in **Fig 1A.** Transformation of *S*. *pneumoniae* was performed as previously described [31]. PCR and Sanger sequencing was conducted on all mutant strains, including the flanking DNA regions, to confirm the presence of the correct DNA sequence (Eton Biosciences).

**Table 1:** Relevant strains and primers used in this study.

Strain	Description		Source
TIGR4	Wild-type serotype 4 encapsulated strain, Gent^R^		Laboratory Strain; Ingeborg Aaberge
TIGR4Δ*cps*	Serotype 4 acapsular strain. *Cps* locus replaced with Spec^R^ gene		Laboratory Strain; Andrew Camilli
TIGR4Δ*pepN*	Serotype 4 strain with the *pepN* gene (SP_0797) replaced with a Cm^R^ gene		This work
TIGR4*pepN*FLAG	Serotype 4 strain with a FLAG epitope tag immediately upstream of the stop codon in the *pepN* gene, Sm^R^		This work
TIGR4*pepN*Revertant	Serotype 4 strain expresses the wild-type *pepN* gene in the Δp*epN* genetic background, Sm^R^		This work
**Primer Name**	**Sequence (5' to 3')**		
**For generating ΔpepN mutant**			
F0 Δ*pepN* Sequencing primer		GAGTTTTTTGACGAAGGG	
R0 Δ*pepN* Sequencing primer		CCTGCCCATAGCTATTAA	
F1 Δ*pepN* For DNA construct		GCACATGTCGTTACAGAA	
R1 Δ*pepN* For DNA construct		**CATCAAGCTTATCGATACCG**GCTTGCATAGTTTTCTCC	
F2 Δ*pepN* For DNA construct		**GAAGGTTTTTATATTACAGCTCCAG**TCGAAGCAGTTGTTCTT	
R2 Δ*pepN* For DNA construct		ATCCAAGCCCAAAAATCG	
**For generating *pepN*FLAG mutant**			
F0 *pepN*FLAG For sequencing		TGATCGGAGATGAAATCG	
R0 *pepN*FLAG For sequencing		CAGCTGATGGAATTTCAC	
F1 *pepN*FLAG For DNA construct		CCTTTTAGCGGATTTGGT	
R1 *pepN*FLAG For DNA construct		TTTATCATCATCATCTTTATAATCTGCATTTCCGTATTGAAG	
F2 *pepN*FLAG For DNA construct		GATTATAAAGATGATGATGATAAATAAATAAGCCTAAAATAAAAAGAA	
R2 *pepN*FLAG For DNA construct		GTCCCCAGAAGTTAATCT	

Gent^R^, gentamycin resistant; Spec^R^, spectinomycin resistant; Cm^R^, chloramphenicol resistant; Sm^R^, streptomycin resistant

Bolded text indicates sequences that are homologous to the Cm^R^ cassette

Underlined text indicate the FLAG epitope sequence

### Sub-cellular fractionation of *S*. *pneumoniae*

To isolate purified cell walls and protoplasts, we followed an established protocol as described previously [32], with some modifications. Briefly, 10mL of mid-log cultures (OD_600_ ~0.6) were pelleted, washed once with 1mL 50 mM Tris-Cl, pH 7.5 and resuspended in 100 μL of cell wall digestion buffer (CWDB) containing 50 mM Tris-Cl, pH 7.5, 30% (w/v) sucrose, 1 mg/mL lysozyme, 300 U/μL mutanolysin (both Sigma-Aldrich), 1x protease inhibitor cocktail (Roche). Cells were incubated for at least 2 h at 37°C with rotation. Whole cell lysates (WCL) were prepared by directly adding SDS buffer to samples and boiling them for 10 mins at 100°C. For protoplast and cell wall (CW), samples were centrifuged at 13,000 x g for 10 min. The supernatant, containing the CW, was applied to SpinX 0.22μm spin columns (Sigma) and centrifuged for 1 min at 13,000 x g to remove any contaminating protoplasts. The pellet, containing the protoplasts, was resuspended in 100μL 50mM Tris-Cl pH 7.5. Samples were stored at -20°C until further use.

### Neutrophil elastase-cell wall degradation assays and SDS-PAGE analysis

CW purified from 10^8^ CFU were incubated with 68μM of neutrophil elastase (NE) (Elastin Products Company) or left untreated for 24 h at 37°C with rotation. Equal volumes of samples were boiled for 10 min in SDS sample buffer containing 50mM Tris-HCl, 10% glycerol, 2% SDS, 0.1% bromophenol blue, 2% β-mercaptoethanol and run on an AnykD gradient Tris-glycine polyacrylamide gel (Bio-Rad). Gels were stained with Colloidal Coomassie Blue (Bio-Rad) for 18h and were visualized using an Epson Perfection V550 Photo scanner.

### Protein sequence analysis by LC-MS/MS

To determine the identity of proteins degraded by NE, bands of interest were excised from the gel and analyzed by the Taplin Mass Spectrometry Facility at Harvard University. Gel bands were cut into ~1 mm^3^ pieces and then subjected to a modified in-gel trypsin digestion procedure [33]. Gel pieces were washed and dehydrated with acetonitrile for 10 min, after which the acetonitrile was removed and the gel pieces were then completely dried in a speed-vac. Gel pieces were rehydrated with 50 mM ammonium bicarbonate solution containing 12.5 ng/μl modified sequencing-grade trypsin (Promega, Madison, WI) at 4°C. After 45 min, excess trypsin solution was removed and replaced with 50 mM ammonium bicarbonate solution. Samples were then incubated at 37°C overnight. Peptides were later extracted by removing the ammonium bicarbonate solution, followed by one wash with a solution containing 50% acetonitrile and 1% formic acid. The extracts were then dried in a speed-vac (~1 hr) and the samples were then stored at 4°C until analysis.

For the analysis, samples were reconstituted in 5–10 μl of HPLC solvent A (2.5% acetonitrile, 0.1% formic acid). A nano-scale reverse-phase HPLC capillary column was created by packing 2.6 μm C18 spherical silica beads into a fused silica capillary (100 μm inner diameter x ~30 cm length) with a flame-drawn tip [34]. After equilibrating the column, each sample was loaded onto the column via a Famos auto sampler (LC Packings, San Francisco CA). A gradient was formed and peptides were eluted with increasing concentrations of solvent B (97.5% acetonitrile, 0.1% formic acid).

Eluted peptides were then subjected to electrospray ionization and then entered into an LTQ Orbitrap Velos Pro ion-trap mass spectrometer (Thermo Fisher Scientific, Waltham, MA). Peptides were detected, isolated, and fragmented to produce a tandem mass spectrum of specific fragment ions for each peptide. Peptide sequences, and hence protein identity, were determined by matching protein databases with the acquired fragmentation pattern by the software program, Sequest (Thermo Fisher Scientific, Waltham, MA) [35]. All databases include a reversed version of all the sequences and the data was filtered to between a one and two percent peptide false discovery rate.

### Subcellular localization of PepN via Western blot analysis

CW was isolated from 10^7^ CFU of the appropriate bacterial strains. Equal volumes of samples were analyzed via SDS-PAGE as described above, proteins were transferred to a nitrocellulose membrane (ThermoFisher) at 22V for 18h at 4°C and blocked for 1 h at room temperature with 5% skim milk in 1X PBS with 0.2% Tween-20 (PBST). Unconjugated primary mouse anti-FLAG antibody (Sigma-Aldrich) at 1:3,000 and rabbit anti-CodY serum (a generous gift from Dr. A. L. Sonenshein) at 1:10,000 diluted in 2.5% milk in 1X PBST were added to membranes and incubated for 1 h at room temperature with agitation. Membranes were then washed 3X with 10mL 1X PBST for 5 mins each. HRP-conjugated secondary antibodies (Jackson ImmunoResearch) were applied at a 1:10,000 dilution in 2.5% milk in 1X PBST and incubated for 1 h at room temperature, followed by three 45 min washes in 10mL 1X PBST. Membranes were then developed with SuperSignal West Dura Extended Duration Substrate (ThermoFisher).

### Capsule immunodot blot assays

To quantify the amount of capsule present in wild-type TIGR4 and *ΔpepN* mutant strains, 1 ml of OD_600_-matched mid-exponential-growth-phase bacteria was pelleted and stored at −20°C until use. Samples were resuspended in 300 μl of CWDB, described above, and incubated at 37°C for 30 min. Samples were then sonicated for 4–10 second intervals while on ice using a probe sonic dismembrator (Fisher Scientific). Next, a 2-fold serial dilution of lysate was prepared in 1X PBS, and 5 μl was spotted on 0.2-μm-pore-size nitrocellulose membranes (Invitrogen, Inc.) with suction. Membranes were blocked with 10mL of 5% milk in 1X TBS with 0.1% Tween-20 (TBST) for 1 h at room temperature with shaking, then washed 1X with 10mL 1X TBST for 5 min. The membrane was then probed with an unconjugated rabbit anti-serotype 4 serum (Statens Serum Institut; 1:1000) in 5mL of 2.5% milk in 1X TBST for 1h at room temperature with shaking. After 3–5 min washes with 15mL 1X TBST, an HRP-conjugated goat-anti-rabbit secondary antibody (1:2500, Jackson ImmunoResearch) was applied to the membrane in 5mL of 2.5% milk in 1X TBST for 1h at room temperature with shaking. Finally, the membrane was washed 3X with 10mL 1X TBST, developed using ECL Blotting Substrate (Thermo Scientific), visualized using the C-Digit Western Blot Scanner, and quantified using ImageStudioLite (LI-COR Biosciences).

### C3 deposition assay and FACS analysis

For C3 deposition assays, 1 ml of mid-exponential phase bacteria grown in THY were pelleted, washed in PBS and resuspended in 500 μl of Hanks buffer with Ca^2+^ and Mg^2+^ (Gibco, Corp.) supplemented with 0.1% gelatin (Fischer Scientific, Inc.). 10^7^ CFU in 50 μl were added to a final concentration of 10% infant rabbit serum in 100 μl (AbD Serotec, Co.). Samples were incubated in a 37°C rolling incubator for 30 mins. Next, opsonization reactions were chilled for 3 mins on ice, quenched with 500 μl of Hanks buffer without Ca^2+^ and Mg^2+^ (Gibco) with 0.1% gelatin, and pelleted at 4000 rpm for 5 mins. Pellets were resuspended in 1:200 FITC-conjugated goat anti-rabbit C3 antibody (MP Biomedicals) in 100 μl Hanks buffer without Ca^2+^ and Mg^2+^ with 0.1% gelatin and incubated on ice in the dark for 30 mins. Staining reactions were quenched with 500 μl Hanks buffer with 0.1% gelatin but without Ca^2+^ and Mg^2+^, centrifuged at 4000 rpm for 5 mins, and pellets were resuspended in 300 μl of 1% paraformaldehyde (PFA; Sigma-Aldrich, Co.). Samples were analyzed on an Se3 Cell Sorter (Bio-Rad) and data were analyzed using FlowJo software.

### *In vitro* bactericidal and PepN degradation assays with neutrophil elastase

Wild-type and mutant strains of *S*. *pneumoniae* were grown to early-exponential phase (OD_600_ ~0.3) in TSB supplemented with catalase, pelleted by centrifugation, washed in sterile 1X PBS, and resuspended in 10 μM sterile sodium phosphate reaction buffer to a concentration of ~10^7^ CFU/mL. 50μL of cells were exposed to a two-fold dilution series of NE ranging from 3.4–13.6μM and incubated for 1h at 37°C with 5% CO_2_. These NE concentrations are within the documented physiological range observed *in vivo* [36] and are similar to concentrations tested by other groups [9, 37]. To determine viable counts, cells were then serially diluted, plated on TSA and incubated overnight at 37°C with 5% CO_2_.

To assess whether PepN is degraded by NE in the context of an intact *Spn* cell, ~5 x 10^6^ CFU were exposed to 6.8μM NE, or left untreated, for 1h at 37°C with 5% CO_2_, followed by the isolation of CW and WCL fractions as described above. Samples were then subjected to SDS-PAGE and Western Blot analysis using anti-FLAG or anti-CodY antibodies. Data were compiled from at least three independent experiments.

### *Ex vivo* neutrophil opsonophagocytic killing assay

These studies were approved by the Human Investigation Review Board (IRB) at both Tufts Medical Center and the University of Buffalo. Young, healthy human volunteers were recruited in accordance to University of Buffalo and Tufts Medical Center IRB and signed informed consent forms. Individuals taking medication, reporting symptoms of infection within the last two weeks, or who were pregnant were excluded from the study. 20mL of whole blood was obtained and anticoagulated with acid citrate/dextrose. PMNs were isolated using a 2% gelatin sedimentation technique as previously described [38] which allows for isolation of active PMNs with ~90% purity. Killing of opsonized *Spn* by human neutrophils was performed as previously described [39] based on a modified protocol described by Dalia *et al*. [40]. Strains of *Spn* were grown to mid-log and 10^3^ CFU were opsonized in 10% (v/v) baby rabbit serum (Pel-Freeze) in 200μL of Hank’s Buffer supplemented with 0.1% gelatin for 30 mins. Pre-opsonized bacteria were then mixed with 5 x 10^5^ PMNs for 45 mins at 37°C with rotation. Samples were then placed on ice to stop the process of opsonophagocytosis followed by serial dilution and plating to enumerate viable CFU. The percentage of bacterial killing was calculated relative to controls without PMNs. In some experiments, neutrophils were treated with 20μM cytochalasin D (Sigma-Aldrich) for 30 min at 37°C prior to incubation with opsonized *Spn* cells [9].

### Statistical analysis

To determine if a statistically significant difference existed between wild-type TIGR4 and the mutant strain of interest across various concentrations of NE, two-way ANOVA statistical tests were performed [41]. In experiments that compared the phenotypes of wild-type TIGR4, *ΔpepN* and *ΔpepNRev* strains in the presence of only 3.4 μM NE, a one-way ANOVA followed by a multiple comparisons test was performed. Unpaired Student t-test was used for comparison of bacterial killing by PMNs. *p* values less than 0.05 were considered significant. All statistical analyses were performed using GraphPad Prism for Mac (GraphPad Software, Inc).

## Results

### Analysis of purified CW from TIGR4 treated with the NSPs, neutrophil elastase (NE) or cathepsin G (CG)

**Fig 2:**
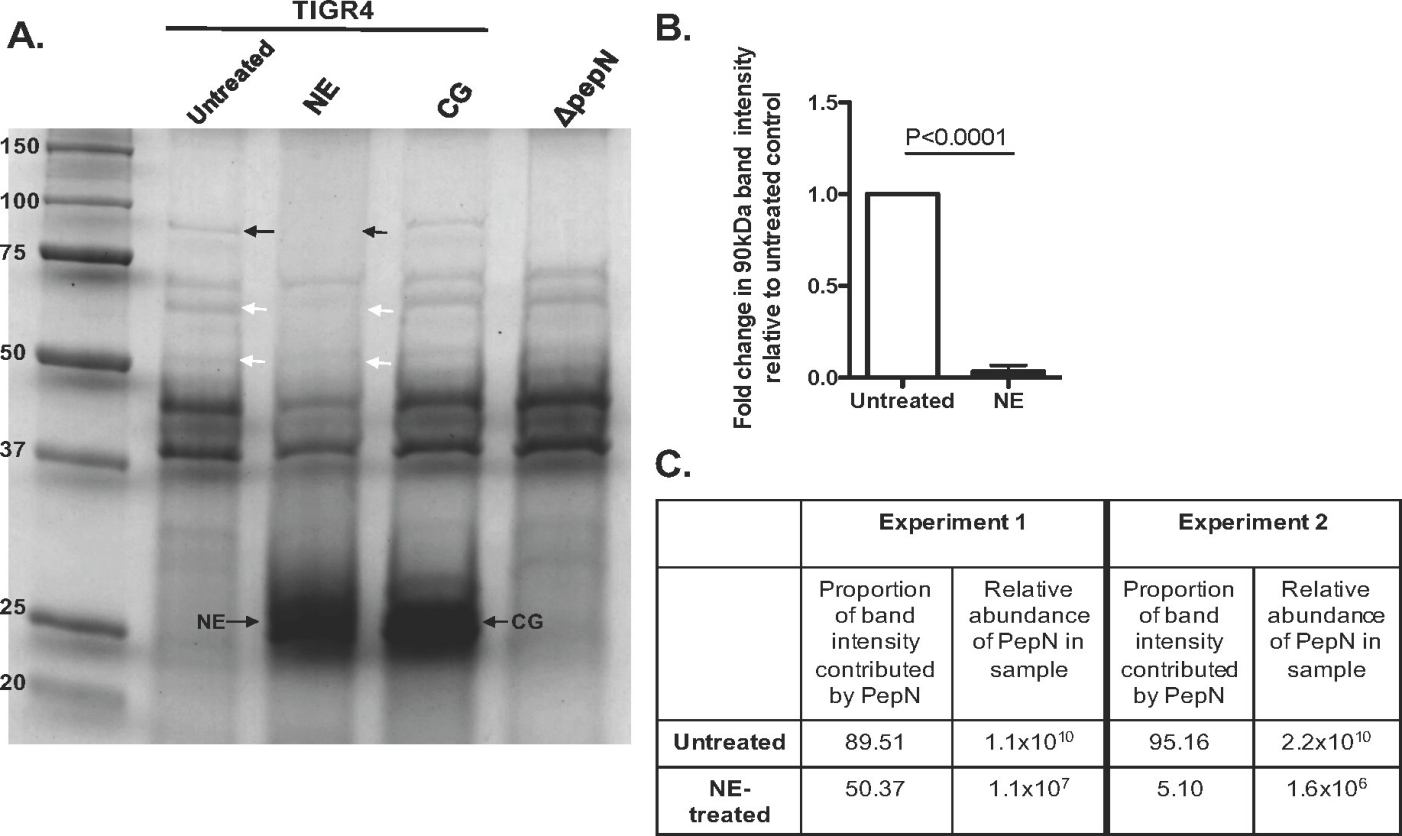
Neutrophil elastase degrades a 90kDa *Spn* CW protein *in vitro*. CW was isolated from TIGR4 and Δ*pepN* cells of *S*. *pneumoniae* and were left untreated or incubated with 68μM NE or 10μM CG. **(A)** Samples were analyzed using an AnykD gradient Tris-glycine polyacrylamide gel followed by colloidal Coomassie blue staining. Arrow heads indicate bands degraded by NE. Black arrow heads highlight the 90kDa species degraded by NE. **(B)** The intensity of the 90 kDa band in the untreated control or in CW samples treated with NE was quantified using ImageStudioLite. The data are presented as relative band intensity normalized to a blank lane. Data shown are means ± SD from 5 independent experiments. P = 0.019 using a Student’s t-test. **(C)** To determine the identity of the 90kDa protein that is degraded by NE, we excised this band from both the untreated and NE-treated lanes and had it analyzed via mass spectrometry. Additionally, this analysis quantified the relative abundance of the 90kDa protein in both the untreated and NE-treated samples. The gel is from one experiment representative of 5 independent experiments. The mass spectrometry analysis is from 2 of those independent experiments. NE, neutrophil elastase; CG, cathepsin G.

Although NSPs were demonstrated to be essential for the effective killing of *Spn* [9], the protein targets that are degraded by these enzymes remains unknown. To identify *Spn* CW proteins that are degraded by NSPs, CW was isolated from wild-type TIGR4 bacteria and were incubated with NE, CG or left untreated. Samples were then evaluated using SDS-PAGE analysis followed by staining with Coomassie blue. As depicted in **Fig 2A** and highlighted by the arrow heads, several protein species (~90kDa, ~70kDa, ~52kDa) were degraded by NE, but were still clearly visible in both the untreated and CG-treated samples. In particular, there was a significant reduction in the intensity of a ~90kDa band **(Fig 2A, black arrow heads)** in the NE-treated samples as compared to the untreated control **(Fig 2B,** P<0.0001**)**. In order to determine the identity of this specific NE substrate, we excised the 90kDa band from the gel in both the untreated and NE-treated samples for mass spectrometry analysis. Based on its amino acid sequence, this protein was determined to be aminopeptidase N (PepN; SP_0797). Importantly, this analysis also revealed that the 90kDa band was vastly comprised of PepN, ranging from ~89–95% of the total band intensity **(Fig 2C)**. Additionally, by comparing the relative abundance of the PepN protein in the untreated versus NE-treated samples, we observed that PepN was substantially degraded, with up to a 10^4^-fold reduction in protein abundance **(Fig 2C)**. Further confirming that the vast majority of the 90kDa band was comprised of PepN, CW isolated from a mutant strain of *Spn* that lacks PepN (Δ*pepN*) did not possess the 90kDa band **(Fig 2A)**. Taken together, these data demonstrate that PepN is a 90kDa protein found in a purified *Spn* CW preparation that is specifically and markedly degraded by NE.

### Characterization of PepN as a CW-localized protein in *Spn* that is degraded by NE *in vitro*

**Fig 3:**
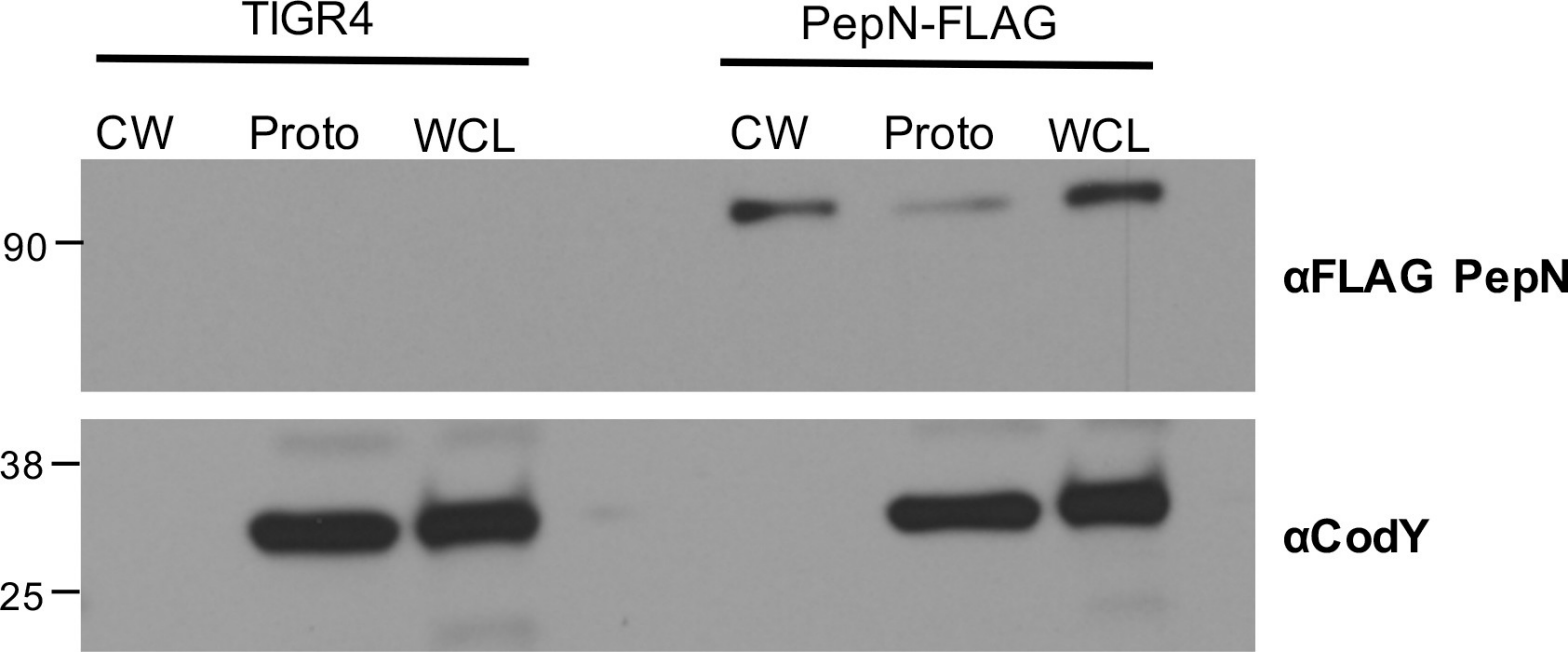
PepN localizes to the cell wall compartment within *Spn* cells. Whole cell lysates (WCL), CW and protoplast fractions were isolated from OD_600_-matched, mid-log phase TIGR4 and PepNFLAG cells. Samples were analyzed by Western blotting using anti-FLAG or anti-CodY (a cytoplasmic protein) antibodies. Data shown are from one experiment representative of three independent experiments.

Based on its amino acid sequence, PepN is annotated as a member of the peptidase M1 family and possesses an active site that is coordinated by three zinc binding sites **(Fig 1B)**. However, examination of the PepN amino acid sequence failed to reveal a canonical LPxTG motif, choline binding domain, Sec secretion signal peptide, or any other characterized export signal [42]. While it is not impossible for a protein lacking a classic export sequence to be secreted from the cell, such as the Pht proteins and those described as non-classical surface proteins [42, 43], this prompted a more thorough investigation of the sub-cellular localization of PepN. To do so, we generated a mutant strain of *Spn* via natural transformation using a DNA construct that added a FLAG epitope immediately upstream of the stop codon **(Fig 1A; *pepNFLAG*)**. For these experiments, we prepared CW, protoplasts and whole cell lysates (WCL) from mid-exponential phase TIGR4 and *pepNFLAG* cells and analyzed these fractions via Western Blotting using anti-FLAG or anti-CodY (a cytoplasmic protein) antibodies. As expected, CodY was detected only in the protoplast and WCL fractions, and was completely absent in the CW samples, confirming that this fraction was free of cytoplasmic contaminants **(Fig 3A, bottom panel).** Importantly, the PepNFLAG protein was clearly detected in the both the WCL and CW fractions, with a fainter band present in the protoplast sample **(Fig 3A, top panel)**. To confirm that observed variations in the PepNFLAG signal were not due to differences in the amount of total protein, we analyzed the CW, protoplast and WCL samples in parallel via SDS-PAGE and Coomassie blue staining. Indeed, similar amounts of protein were observed in the protoplast and WCL samples isolated from TIGR4 and PepNFLAG cells, while the CW fraction isolated from both strains contained the least amount of protein **(S1 Fig).** Thus, the intensity of the PepNFLAG signal in the CW fraction **(Fig 3A, top panel)** may possibly represent an underestimation of the amount of PepN protein in this compartment. Together, these data suggest that despite the absence of a canonical LPxTG motif, Sec-dependent signal peptide, or any known cell wall binding motif, PepN indeed localizes to the CW compartment.

### Neutrophil elastase degrades PepN in intact *Spn* cells

**Fig 4:**
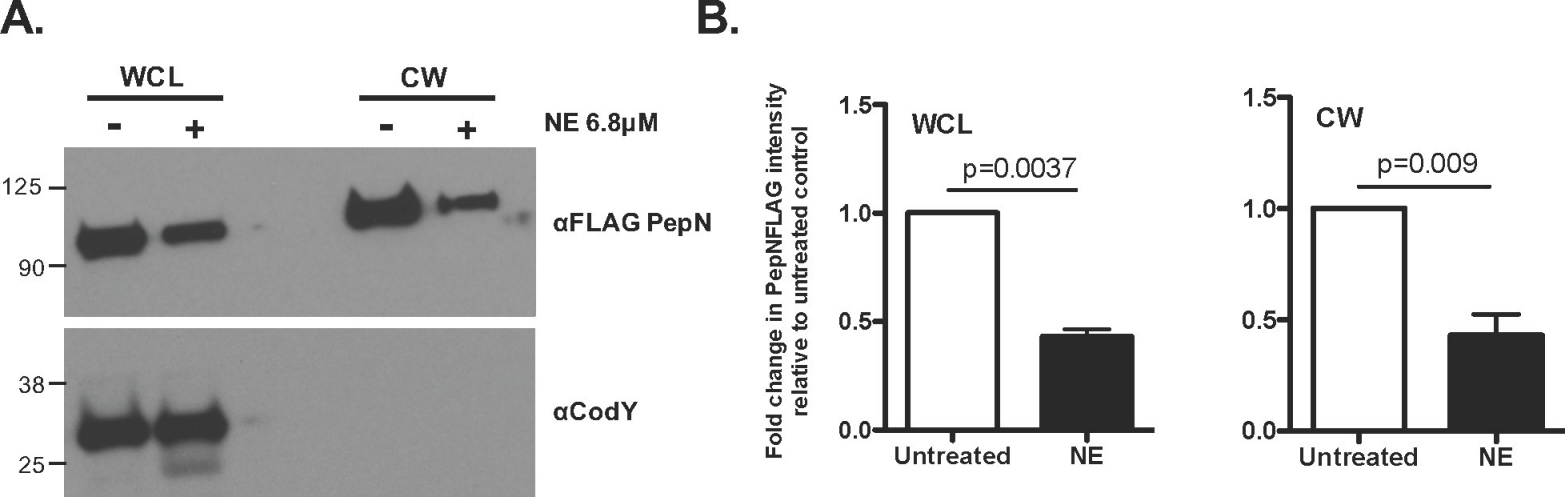
NE degrades PepN in intact *Spn* cells. Approximately 5 x 10^6^ CFU of the PepNFLAG strain were grown to early-log phase and were either left untreated or were exposed to 6.8μM NE. **(A)** Cells were fractionated to isolate the CW and samples were analyzed via Western blotting using anti-FLAG or anti-CodY (a cytoplasmic protein) antibodies. Data shown are from one Western blot representative of four independent experiments. (**B)** Band intensity in CWL and CW samples were normalized to a blank lane, are expressed as fold change relative to the untreated control and were quantified using ImageStudioLite software. Data presented are the means ± SD from 4 independent experiments.

To validate PepN as the substrate for NE and confirm that NE can degrade PepN in the context of native cell wall architecture, we exposed PepNFLAG cells to NE, followed by subcellular fractionation and Western Blot analysis using anti-FLAG or anti-CodY antibodies. Again, the CodY signal was only present in the WCL samples and the intensity of this band was unchanged in the presence of NE **(Fig 4A, bottom panel)**. Strikingly, analysis of both WCL and CW fractions revealed a strong PepNFLAG signal in the untreated samples **(Fig 4A, top panel)** that was diminished >2-fold upon exposure to NE (WCL P = 0.0037; CW P = 0.009) **(Fig 4B)**. Importantly, quantification of total protein loaded in untreated or NE-treated WCL and CW samples were evaluated via SDS-PAGE analysis and Coomassie blue staining. These data confirm that, overall, there were no significant differences in protein content among these samples **(S2 Fig).** These data strongly suggest that in the context of an intact *Spn* cell, PepN is a CW-localized protein that is significantly degraded by NE.

### The Δ*pepN* mutant demonstrates enhanced resistance to killing by purified NE *in vitro* and by human neutrophils *ex vivo*

**Fig 5:**
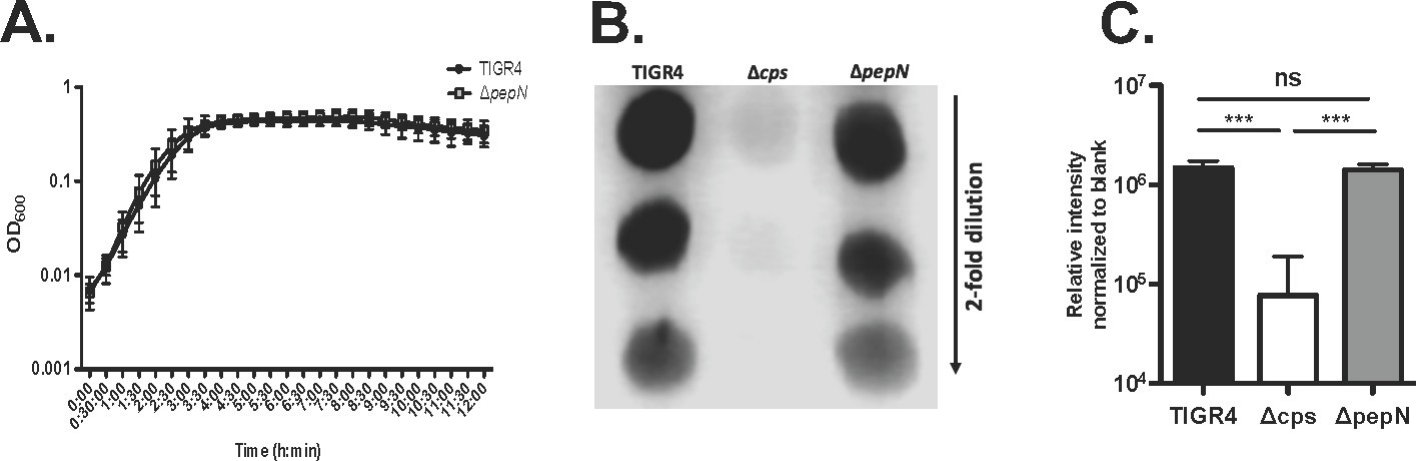
Mutants lacking PepN (Δ*pepN)* demonstrate the same growth kinetics and capsule levels as wild-type TIGR4. **A)** TIGR4 and Δ*pepN* cells were grown to mid-log phase, back-diluted in fresh media to OD_600_ 0.003, incubated at 37°C and OD_600_ was measured every 30 minutes for 12h. Data presented are the means ± SD from 3 independent experiments. **B and C)** Capsule was isolated from OD_600_-matched, mid-log phase TIGR4, Δ*cps* and *ΔpepN* cells. For each strain, 5μL spots of a 2-fold serial dilution were applied to a nitrocellulose membrane, which was developed using an unconjugated rabbit anti-serotype 4 serum, HRP-conjugated goat anti-rabbit antibody and ECL Blotting Substrate. **B)** Data shown are from one blot representative of 3 independent experiments. **C)** Data presented are the means ± SD from 3 independent experiments. ***, P<0.001; ns = no significant difference. Statistical analyses were conducted using a one-way ANOVA and Tukey’s Multiple Comparisons Test.

To assess the contribution of PepN to the effective killing of *Spn in vitro*, we generated a mutant strain lacking this protein (*ΔpepN*). To first characterize this mutant, we assessed its growth kinetic phenotype, and determined that it was similar to that of wild-type TIGR4 (**Fig 5A)**. Additionally, to ensure that removal of the cell wall protein PepN did not have an unexpected impact on capsule level, a structure that was demonstrated to influence susceptibility to NSP-mediated killing [37], we quantified and compared capsule on wild-type and Δ*pepN* cells. As shown in **Fig 5B and 5C,** while wild-type TIGR4 and *ΔpepN* cells both express significantly more capsule than the acapsular control (Δ*cps;* both P<0.001), there was no significant difference in capsule levels between wild-type TIGR4 and the *ΔpepN* strain.

**Fig 6:**
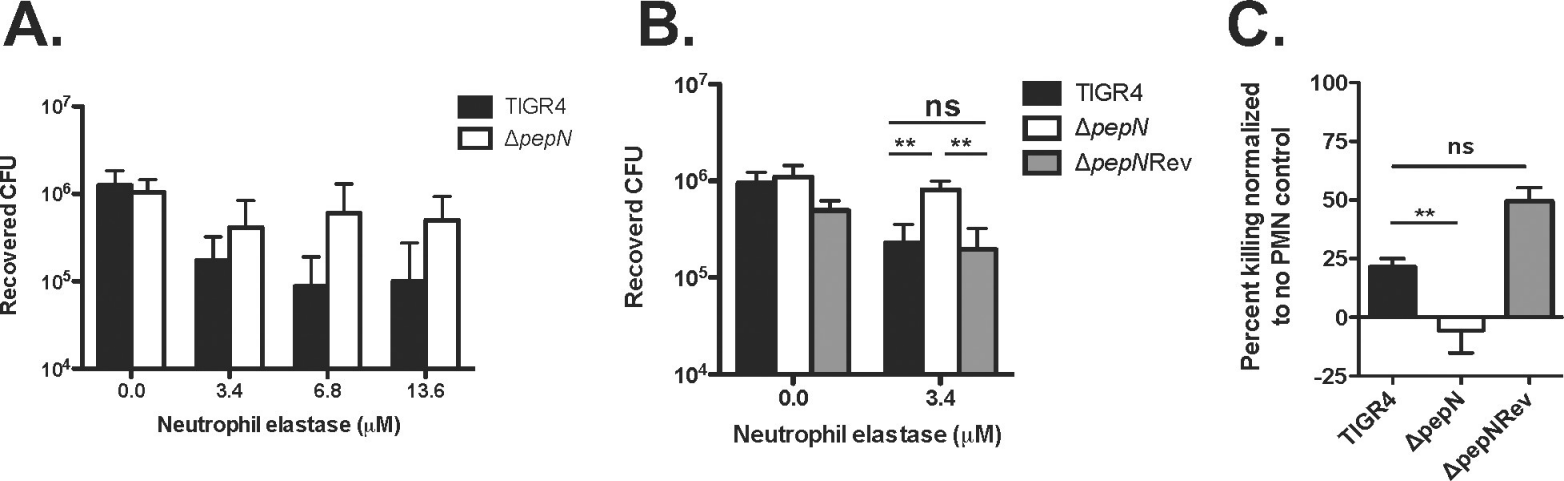
The Δ*pepN* mutant exhibits enhanced resistance to NE-mediated killing *in vitro*. and to killing by human neutrophils *ex viv*o. **A)** Approximately 5 x 10^5^ CFU of the TIGR4 or *ΔpepN* strain were left untreated or exposed to various concentrations of NE. Samples were diluted and plated to enumerate viable CFU. Data presented are the means ± SD from 3–4 independent experiments. P = 0.004 comparing TIGR4 to *ΔpepN* across all concentrations of NE; P = 0.0035 evaluating dose dependent effect of NE-mediated killing within each individual strain. Statistical analyses were calculated using two-way ANOVA. **B)** TIGR4, Δ*pepN* and *ΔpepN*Rev cells were left untreated or were exposed to 3.4μM NE followed by serial dilution and plating to enumerate viable CFU. Data presented are the means ± SD from 3 independent experiments. **, P<0.01; ns = no significant difference. P values were calculated using one-way ANOVA. **C)** For opsonophagocytic killing experiments, PMNs were isolated from the blood of healthy donors and incubated with pre-opsonized *Spn*. Viable CFU were determined after serial dilution and plating. The percentage of bacteria killed was determined by comparing surviving CFU for each strain to a no PMN control. Positive percent killing indicates bacterial death while negative percent indicates bacterial growth. Data are shown as the means ± SD from 4 independent experiments with PMNs from four separate donors. P = 0.036 and was calculated using Student’s t-test.

**Fig 7:**
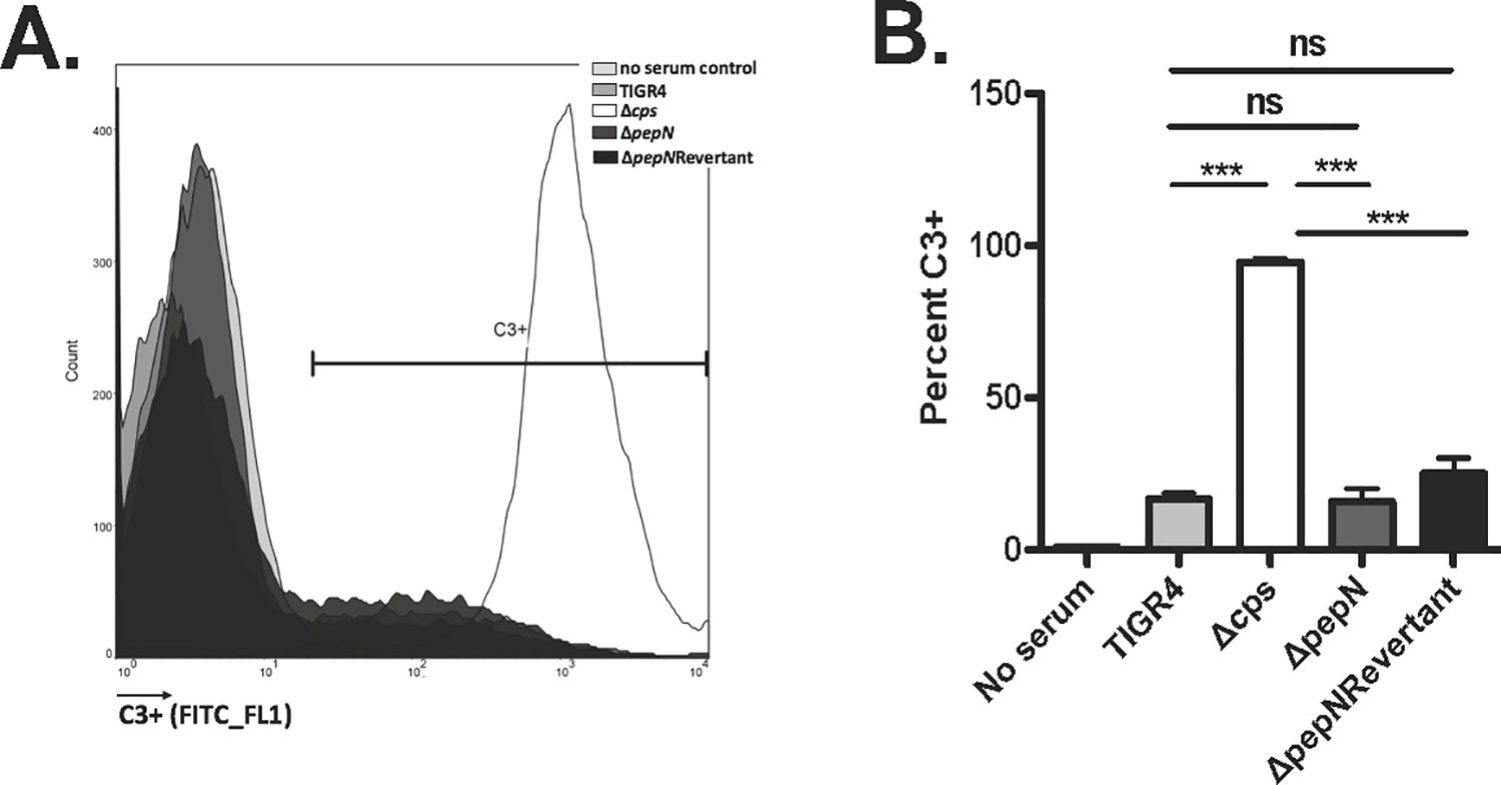
Mutants lacking PepN demonstrate the same C3 deposition phenotype as wild-type TIGR4. *Spn* cells were incubated with infant rabbit serum and stained with a FITC-conjugated anti-C3 antibody. Data are shown as **A)** a representative histogram plot from 1 experiment representative of 3 independent experiments and **B)** means ± SD from 3 independent experiments. ***, P<0.001; ns = no significant difference. P values were calculated using one-way ANOVA.

In subsequent experiments, we assessed the phenotype of the *ΔpepN* strain in the presence of various concentrations of purified NE. These data demonstrated that NE killed both wild-type TIGR4 and *ΔpepN* strains in a significant, dose-dependent fashion (P = 0.0035). Importantly, the *ΔpepN* mutant was significantly more resistant (P = 0.004) to NE-mediated killing as compared to wild-type TIGR4 across all tested concentrations of NE (**Fig 6A and 6B)**. Furthermore, as shown in **Fig 6B,** when we reverted the *ΔpepN* mutant (*ΔpepN*Rev) and exposed this strain to purified NE, it exhibited a sensitive phenotype similar to that of wild-type TIGR4. To test the *ΔpepN* mutant in a more physiologically relevant setting, we determined its survival phenotype using an opsonophagocytic assay with purified human neutrophils. These experiments revealed that, compared to wild-type *Spn*, the *ΔpepN* mutant was also significantly more resistant (P = 0.036) to extracellular phagocytic killing by human neutrophils **(Fig 6C)**. In fact, while wild-type TIGR4 was killed in the presence of PMNs, the *ΔpepN* mutant was not and displayed a very slight overall increase in cell number **(Fig 6C)**. Importantly, the resistance of the *ΔpepN* strain to neutrophil-mediated phagocytosis was not due to differences in neutrophil viability **(S3 Fig)** nor opsonization by complement C3, a protein vital to the uptake of *Spn* via the classical complement pathway [44, 45]. As shown in **Fig 7A and 7B,** there was no significant difference in the percent of C3+ *Spn* cells between the WT, *ΔpepN* and *ΔpepN*Rev strains. Taken together, these data reveal that PepN is a nonessential, cell wall-localized protein that is a substrate of NE, and its degradation contributes to the effective killing of *Spn* by both purified NE and whole human neutrophils.

## Discussion

In neutrophils, non-oxidative mechanisms of killing phagocytosed *Spn* were shown to be essential both *in vitro* and *in vivo* and primarily involve the activity of NSPs, such as NE and CG [9, 10]. Further supporting the importance of NSPs, individuals with impaired granule mobilization, which affects the fusion of NSP-containing granules with the phagolysosome, exhibit a defect in neutrophil-mediated killing of *Spn* [12]. In addition to playing a key role in controlling *Spn* infections, other studies demonstrated that NSPs have the capacity to directly kill or degrade virulence factors produced by a variety of pathogens including *P*. *aeruginosa*, *E*. *coli*, *S*. *flexneri*, *K*. *pneumoniae* and *S*. *aureus* [20–25]. Although previous reports highlight the importance of NSPs in bacterial clearance, little is known about the identity of the *Spn* proteins that are degraded by NSPs and thus facilitate effective killing.

In this study, we aimed to identify *Spn* CW proteins that are degraded by the NSPs NE and CG. To do so, we exposed a purified *Spn* CW preparation to NE and CG *in vitro*. These experiments revealed that a few bands, ~90kDa, ~70kDa and ~52kDa in size, were degraded only by NE, suggesting a difference in substrate specificity between these two NSP family members. Since over multiple independent experiments, the ~90kDa band was the most strikingly degraded by NE in multiple independent experiments, this study focused on further identifying and characterizing this CW protein as a putative NE substrate and determining its potential role in NSP-mediated killing of *Spn*. Subsequent analysis via mass spectrometry revealed this ~90kDa protein to be aminopeptidase N (PepN). Furthermore, these experiments demonstrated that the relative abundance of PepN was reduced 10^3^−10^4^-fold in purified CW samples treated with NE as compared to untreated controls, indicating that NE substantially degrades this *Spn* protein. Interestingly, in the context of an intact *Spn* cell, PepN abundance was more modestly reduced (~3-fold) in NE-treated samples, as compared to untreated controls. This discrepancy may be due, in part, to the masking effect provided by the capsular polysaccharide and/or the complex architecture of the cell wall, thus limiting the accessibility of PepN as a NE substrate. Nevertheless, degradation of PepN on intact *Spn* cells was still possible and thus may be sufficient to induce bacterial cell death.

Since analysis of the PepN sequence failed to reveal a canonical Sec-dependent secretion signal, LPxTG cell wall anchoring motif or other export signals [42, 43], we created a mutant strain of *Spn* harboring a FLAG-tagged version of the protein to more definitively assess its subcellular localization. From these experiments we visualized a faint PepN band in the protoplast fraction. It is still unclear how PepN is exported from the cell, therefore these data would suggest that the export mechanism is rate-limiting and may depend on there being a sufficient concentration of PepN in the cytoplasm. However, due to the absence of a Sec-dependent signal sequence, it seems unlikely that coupled translation and secretion is occurring.

Interestingly, despite the absence of an obvious secretory or CW localization motif, the majority of PepN was identified in the CW compartment. Although these findings are at odds with the reported cytoplasmic localization of PepN in *S*. *mitis* (97% sequence identity) and *S*. *salivarius* (67% sequence identity) [46, 47], the absence of the cytoplasmic control protein CodY in our CW fractions provides confidence that PepN is indeed CW localized in *Spn*. Additionally, in various other Gram-positive species including *L*. *lactis*, PepN (57% identity) is a CW anchored protein, indicating there is some variability in the localization of this protein [48]. Thus, akin to other *Spn* proteins including pneumolysin [32], PavA [49] and HtrA [42], our data indicate that PepN is yet another example of a non-classical protein that localizes to the CW despite the absence of an obvious export sequence.

These initial experiments strongly suggest that PepN is a CW-localized protein that serves as a substrate for NE. However, these conclusions were drawn from experiments using a purified preparation of CW proteins, which may affect the abundance, diversity or availability of substrates for NE-mediated degradation. More importantly, since the aim of this study was to identify *Spn* proteins that not only were degraded by NSPs, but were also involved in NSP-mediated killing of *Spn*, we conducted additional experiments in a more physiologically relevant system. Through experiments that exposed intact *Spn* cells expressing the FLAG-tagged version of PepN to NE, we confirmed that PepN does indeed localize to the CW and it is markedly degraded compared to untreated controls. Previous studies in *E*. *coli* and *P*. *aeruginosa* identified the outer membrane proteins OmpA and OprF, respectively, as targets that are degraded by NE that results in bacterial cell death [22, 24]. Both of these proteins are porins that contribute to virulence and isogenic mutants lacking either OmpA or OprF in the respective strain demonstrated enhanced resistance to NE-mediated killing *in vitro* and in relevant mouse models of infection [22, 24, 50, 51]. To directly assess the contribution of PepN to NSP-mediated killing of *Spn* cells, we created the Δ*pepN* strain and confirmed that its growth rate was indistinguishable from that of wild-type *Spn*. Importantly, since capsule level was shown to impact killing by NSPs [37], and it was feasible that the deletion of a CW-localized aminopeptidase could affect the attachment of capsule to the cell, we quantified capsule on wild-type TIGR4 and Δ*pepN* cells. Data from these experiments demonstrated that both strains possess similar amounts of capsule. *In vitro* bactericidal assays that exposed wild-type TIGR4 and Δ*pepN* cells to purified NE revealed that cells lacking PepN were significantly more resistant to killing. Additionally, Δ*pepN* cells were significantly more resistant to opsonophagocytic killing by whole human neutrophils *ex vivo*. Importantly, the differential killing phenotype was not due to variations in capsule level or C3 deposition. Rather, these data suggests that differential killing between wild-type and Δ*pepN* may be due to the absence of a NE substrate that is necessary for optimal killing of *Spn* once in the phagolysosome. Our observation that NE exhibits bactericidal activity with TIGR4 (serotype 4) strains *in vitro* are at odds with a study by Domon *et al* that report an absence of NE-mediated killing in D39, a serotype 2 strain of *Spn* [52]. These differences may be due, in part, to variations in the repertoire or expression level of surface-associated proteins between these two *Spn* serotypes [53]. However, another study by van der Windt and colleagues reported substantial NE-mediated killing of both TIGR4 and D39 strains *in vitro* [37]. Thus it may be possible that other factors, including slight technical variations in experimental parameters, might be responsible for the differences in outcome.

A key observation from these experiments was that NE-mediated degradation of a non-essential CW protein, PepN, may play an important role in *Spn* killing. One possible explanation for this observation is that degradation of PepN is sufficient to destabilize the cell envelope and induce cell lysis. Alternatively, if PepN is attached, in some fashion, to peptidoglycan, teichoic or lipotechoic acid, its degradation by NE may also damage these essential structures or impair their turnover such that normal *Spn* growth is disrupted. Another potential explanation may be that, via its aminopeptidase activity, PepN modifies other CW proteins and creates additional NE substrates. Thus in PepN-sufficient *Spn* cells, not only is PepN directly degraded, but also the modified CW proteins created via PepN aminopeptidase activity may also be degraded, which together is enough to cause cell death. It would be possible to test this last hypothesis by creating a mutant strain with an inactivated PepN catalytic site and assess its viability in the presence of NE. Additionally, since our initial experiments identified three protein bands that were notably degraded only by NE, it is feasible that NE may have additional substrates aside from PepN in the *Spn* CW that potentially contribute to bacterial cell death. These questions are beyond the scope of this current study, but emphasize the need for future experiments.

The role of PepN in *Spn* biology and in the context of host infection is not well understood. However, in other closely related species of *Streptococcus*, including *S*. *thermophilus*, PepN is characterized as a 95kDa monomeric, metallo-aminopeptidase that possesses three Zn^2+^ binding sites that coordinates its active site [26, 48]. Based on its sequence homology to other aminopeptidases, it is thought that PepN functions to degrade endogenous proteins and contributes to normal protein turnover as well as to provide free amino acids to be used for metabolic processes [48]. Interestingly, a recent study demonstrated that PepN present in *Spn* lysates dampens the effector function of cytotoxic T lymphocyte by modulating the intracellular TCR signaling cascade and ultimately dampens the production of the pro-inflammatory cytokine, IFN-γ [54]. Together with the findings in our study, these data begin to reveal a previously underappreciated role for PepN in host-pathogen interactions and *Spn* disease pathogenesis. In summary, this is the first report to identify a *Spn* protein, PepN, as a substrate that is degraded by NE and that also plays a key role in NSP-mediated killing of *Spn* both *in vitro* and *ex vivo*. Additionally, we determined that despite the absence of a canonical export sequence, PepN localizes to the CW compartment within *Spn*. We propose a model where *Spn* cells are opsonophagocytosed by neutrophils and are subsequently bombarded with an assortment of lysosome- and granule-derived anti-microbial factors. Based on our data, a key step in this process is the NE-mediated degradation of PepN, which contributes to the effective killing of *Spn*.

## Supporting information

S1 Fig Quantification of total protein loaded in CW, protoplast and WCL samples. CW, protoplast and WCL fractions were isolated from TIGR4 and PepNFLAG cells. The samples analyzed in this experiment are the same as those presented in Fig 3. Samples were analyzed by **(A)** SDS-PAGE followed by Coomassie Blue staining. **B)** The band intensity in each lane was quantified using ImageStudioLite software and the data are expressed as fold change relative to the cell wall digestion buffer (CWDB) control lane. Data shown are from one experiment representative of three independent experiments.
(TIFF)

S2 Fig Quantification of total protein loaded in CW and WCL samples in the presence or absence of NE. **(A)** Total protein content from three independent experiments in untreated and NE-treated WCL and CW samples were evaluated via SDS-PAGE and Coomassie blue staining. **(B)** Data are normalized to a blank lane, expressed as fold change relative to the respective untreated control and were quantified using ImageStudioLite software. Data shown are the means ± SD from three independent experiments. Student’s t-test revealed no significant differences.
(TIFF)

S3 Fig Quantification of neutrophil viability via trypan blue exclusion after exposure to WT and *ΔpepN* cells. PMNs were isolated from the blood of two healthy donors and 5 x 10^5^ cells were incubated with 10^3^ CFU of WT or *ΔpepN* cells. Following a 45-minute incubation, neutrophil viability was determined via trypan blue exclusion and enumeration using a haemacytometer. Each sample was enumerated twice and by two independent individuals. Data shown are the means ± SD from two independent experiments with at least two technical replicates per strain per experiment. One-way ANOVA revealed no significant difference in neutrophil viability after exposure to WT or Δ*pepN* cells.
(TIFF)
