# Supplementary figures and images for: PepN is a non-essential, cell wall-localized protein that contributes to neutrophil elastase-mediated killing of Streptococcus pneumoniae
Source: PLoS One. 2019 Feb 1;14(2):e0211632. doi: 10.1371/journal.pone.0211632 (PMC6358159; doi:10.1371/journal.pone.0211632)

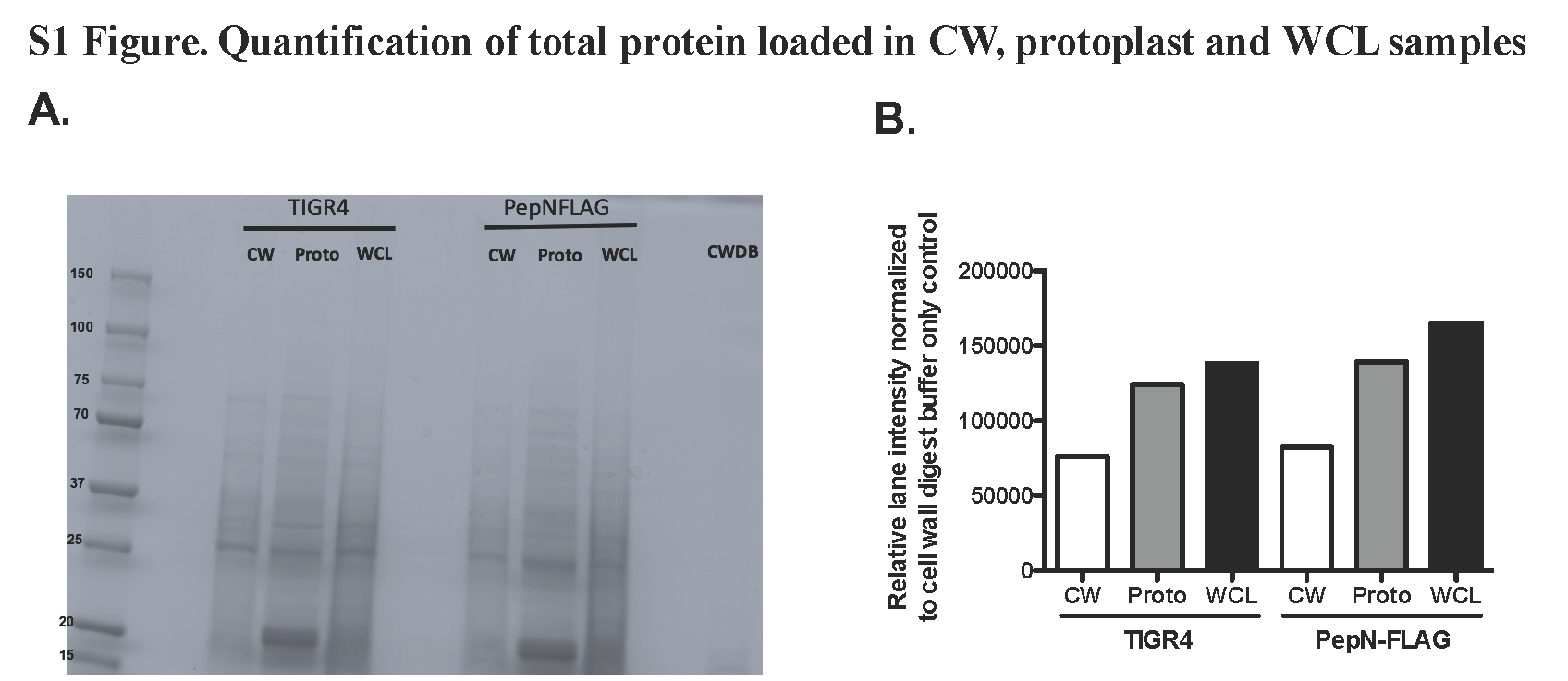

Supplement: S1 Fig — CW, protoplast and WCL fractions were isolated from TIGR4 and PepNFLAG cells. The samples analyzed in this experiment are the same as those presented in Fig 3. Samples were analyzed by (A) SDS-PAGE followed by Coomassie Blue staining. B) The band intensity in each lane was quantified using ImageStudioLite software and the data are expressed as fold change relative to the cell wall digestion buffer (CWDB) control lane. Data shown are from one experiment representative of three independent experiments. (TIFF) [file pone.0211632.s001.tiff]

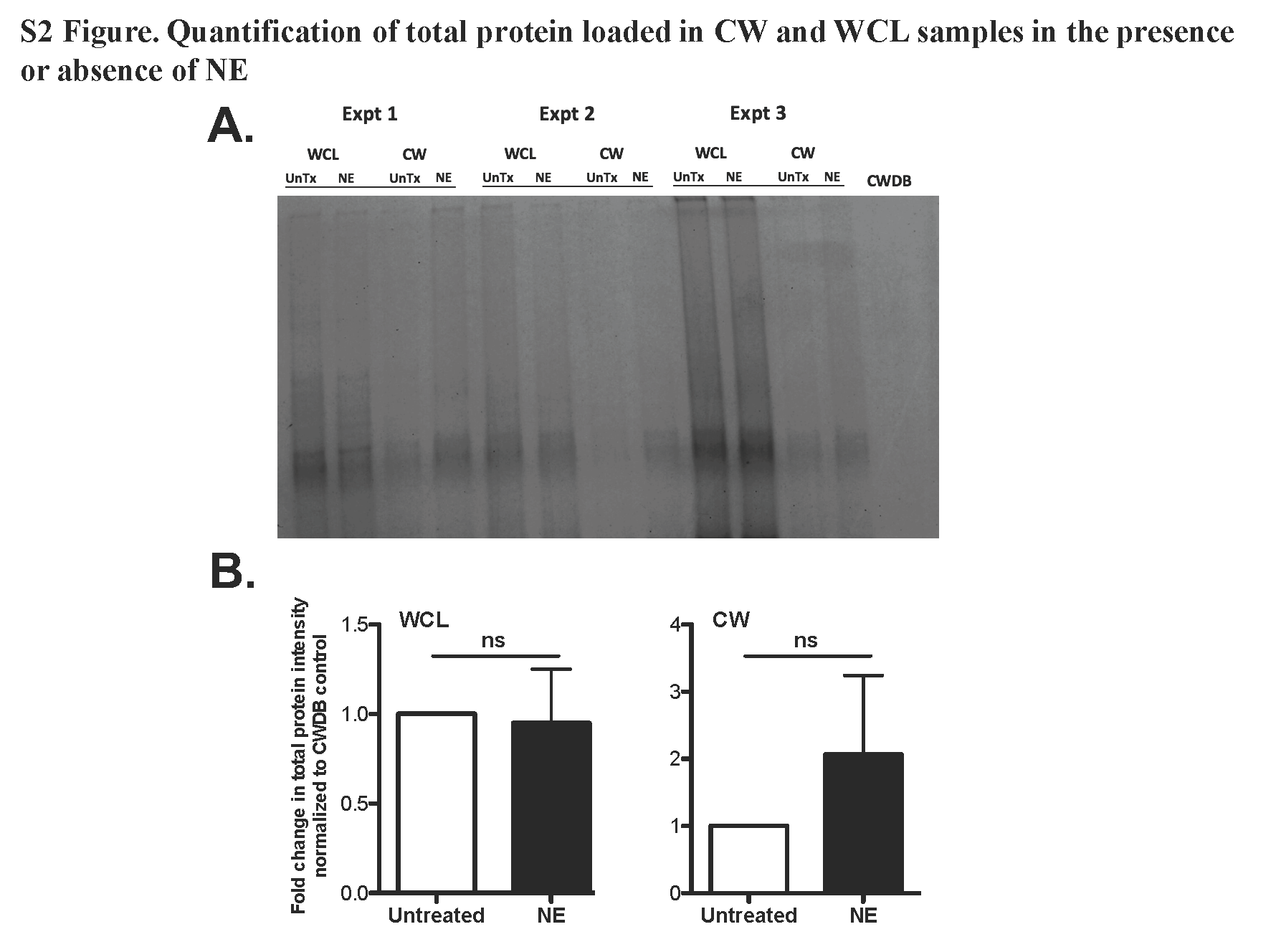

Supplement: S2 Fig — (A) Total protein content from three independent experiments in untreated and NE-treated WCL and CW samples were evaluated via SDS-PAGE and Coomassie blue staining. (B) Data are normalized to a blank lane, expressed as fold change relative to the respective untreated control and were quantified using ImageStudioLite software. Data shown are the means ± SD from three independent experiments. Student’s t-test revealed no significant differences. (TIFF) [file pone.0211632.s002.tiff]

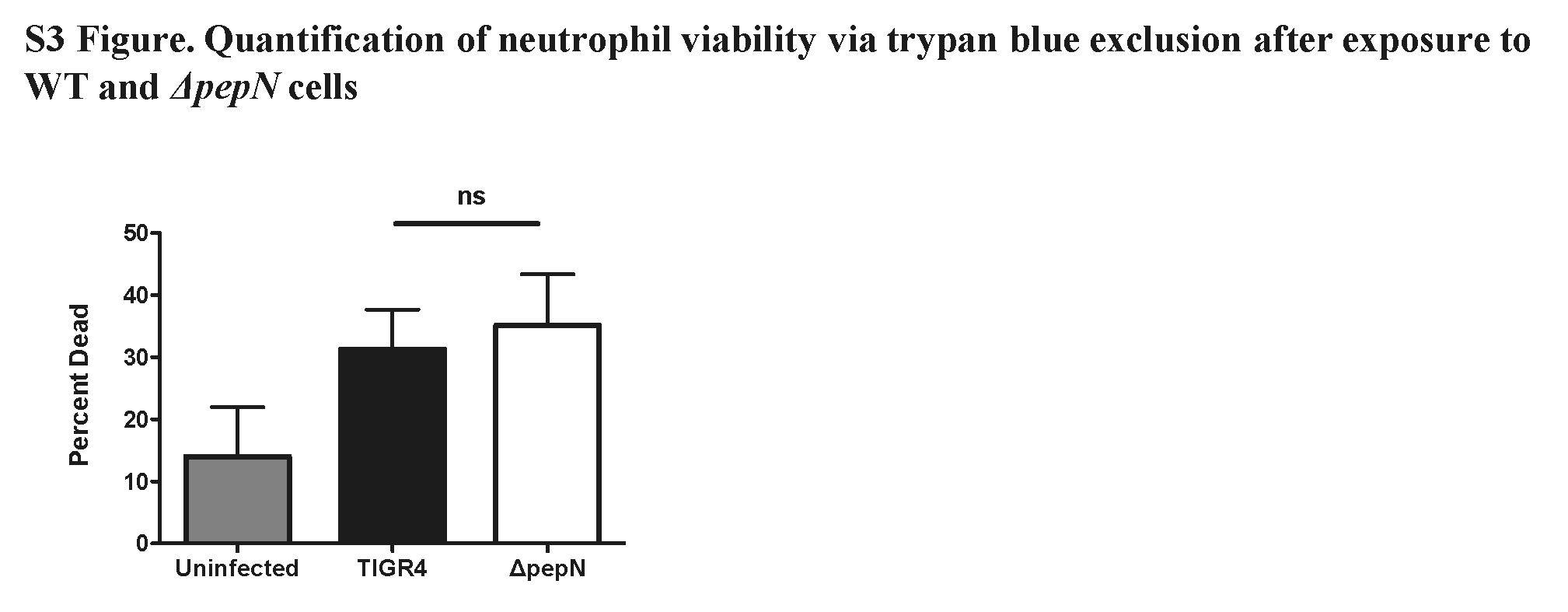

Supplement: S3 Fig — PMNs were isolated from the blood of two healthy donors and 5 x 10^5^ cells were incubated with 103 CFU of WT or ΔpepN cells. Following a 45-minute incubation, neutrophil viability was determined via trypan blue exclusion and enumeration using a haemacytometer. Each sample was enumerated twice and by two independent individuals. Data shown are the means ± SD from two independent experiments with at least two technical replicates per strain per experiment. One-way ANOVA revealed no significant difference in neutrophil viability after exposure to WT or ΔpepN cells. (TIFF) [file pone.0211632.s003.tiff]
